# On the design of a constitutively active peptide asparaginyl ligase for facile protein conjugation

**DOI:** 10.1002/2211-5463.13575

**Published:** 2023-03-28

**Authors:** Niying Chua, Yee Hwa Wong, Abbas El Sahili, Chuan Fa Liu, Julien Lescar

**Affiliations:** ^1^ School of Biological Sciences Nanyang Technological University Singapore City Singapore; ^2^ NTU Institute of Structural Biology Singapore City Singapore

**Keywords:** asparaginyl endopeptidase, peptide asparaginyl ligase, peptide cyclization, protein conjugation, protein refolding

## Abstract

Peptide asparaginyl ligases (PALs) are precision tools for peptide cyclization, cell‐surface labelling, protein semisynthesis and protein conjugation. PALs are expressed as inactive proenzymes requiring low pH activation. During activation, a large portion of the cap domain of the proenzyme that covers the substrate binding site is proteolytically removed, exposing the active site to solvent and releasing a population of heterogenous active enzymes. The availability of a readily active ligase not requiring acid activation and subsequent purification of active forms would facilitate manufacturing and streamline applications. Here, we engineered the *Oa*AEP1b‐C247A hyperactive ligase via serial truncations along the linker connecting the cap and core domain of the proenzyme. The recombinant expression of the truncated constructs was carried out in *Escherichia coli*. Following a solubilization/refolding protocol, one truncated construct termed ‘OaAEP1b‐C247A‐∆351’ could be overexpressed in the insoluble fraction, purified, and displayed a level of ligase activity comparable to the acid‐activated *Oa*AEP1b‐C247A enzyme. This constitutively active protein can be stored for up to 2 years at −80 °C and readily used for peptide cyclization and protein conjugation. We were able to express and purify a stable constitutively active asparaginyl ligase that can be stored for months without significant activity loss. The removal of the low pH proenzyme activation step eliminates the heterogeneity introduced by this procedure. The yield of purified recombinant active ligase that can be routinely obtained per 100 mL of *E. coli* cell culture is about 0.9 mg. This recombinant active ligase can be used to carry out protein conjugation.

## Graphical abstract

Precision tools are needed for the production of homogeneous biologics such as antibody‐drug conjugates or nanobody conjugates for diagnostics and therapy. Peptide asparaginyl ligases are fast and efficient enzymes, but they are expressed as proenzyme and require an acid‐activation step, which introduces heterogeneity. Here, we engineered a constitutively active enzyme that can be overexpressed in *Escherichia coli* and used for bioconjugation.

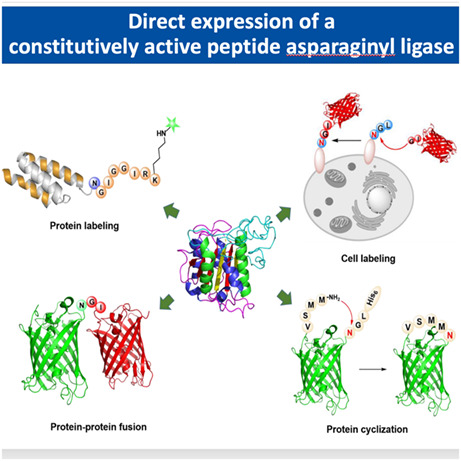

**Abbreviations**
AEP: asparaginyl endopeptidase
FRET: fluorescence resonance energy transfer
PAL: peptide asparaginyl ligase

Enzyme‐mediated peptide ligation [1, 2] has been exploited for a wide range of applications such as protein/peptide ligation, cyclization and labelling, protein thioester formation [3], protein conjugation to various moieties, such as PEG, lipids or fluorescent probes, live‐cell‐surface labelling [4], nanobody conjugation [5], and antibody‐drug conjugation [6, 7, 8, 9, 10, 11]. Since its discovery, sortase A has been a popular choice to perform protein conjugation [12], but a significant amount of enzyme is required often approaching 1 : 1 molar ratio with the target protein. A rather large LPXTG tag must be genetically added to the target protein and the reaction catalyzed by sortase A is reversible.

Asparaginyl endopeptidases (AEPs) and peptide asparaginyl ligases (PALs) were discovered in cyclotide‐producing plants, and both enzymes belong to the cysteine protease family C13. AEPs hydrolyze the Asx‐Xaa peptide bond (Asx is Asn or Asp) at the P1 position of the polypeptide substrate [13, 14]. By contrast, PALs catalyze peptide bond formation. The discovery of hyperactive PALs such as butelase‐1 [11] or *Vy*PAL2 [15] and the engineering of the single mutant *Oa*AEP1b‐C247A [16, 17] has opened possibilities not envisioned before in the field of bioconjugation. Thus, their discovery has attracted intense research activities to facilitate the usage of PALs for various applications in biotechnology and medicine [1, 2].

Structural studies revealed that PALs and AEPs [18, 19, 20, 21, 22] share a similar overall fold formed by a core domain linked to a C‐terminal cap domain via a flexible linker. The core domain consists of a six‐stranded β‐sheet surrounded by six α‐helices located at its periphery, while the cap domain is formed by a suite of α‐helices [17, 23, 24]. An evolutionarily conserved glutamine residue at the N‐terminus of the α6‐helix of the cap (Gln347 in *Oa*AEP1b), inserts into the S1 pocket, keeping the proenzyme in an inactive state [17]. Upon activation at acidic pH values ranging from 4.0 to 4.5, the cap domain becomes separated from the core domain via electrostatic repulsion, facilitating cleavage *in trans* and exposing the enzyme active site to the solvent [19, 25, 26, 27]. This cleavage allows binding by the PAL of polypeptide substrates containing the N/DX1X2 tripeptide motifs, where X1 is any residue besides Pro and X2 is a hydrophobic residue [26]. Such motifs are present at the N‐terminus and within the linker region and cap domain of the proenzyme accounting for autoproteolysis activity observed at these sites. At acidic pH, hydrolysis is favored, leading to the degradation of the cap domain and the N‐terminus of the core domain. *In vivo*, acidic proteolytic activation occurs in the vacuole of cyclotide‐producing plants and serves to regulate the activity of these enzymes endowed with proteolytic and cyclization activity [27, 28, 29, 30, 31].

So far, all PALs reported were expressed recombinantly as zymogens and their enzymatically active isoforms were only obtained following incubation at low pH [17, 21, 24]. Significant heterogeneity is introduced during this low pH activation stage due to the presence of several closely spaced cleavage sites in the proenzyme and the various isoforms are subsequently difficult to separate via chromatography. Thus, the auto‐activation process yields a mixture of heterogeneous activated forms of ligases due to the presence of multiple accessible activation sites at both the N and C termini of the proenzyme. This heterogeneity could be an issue for various industrial applications from a manufacturing and quality control perspective. To eliminate the time‐consuming low pH activation step of the proenzyme and address the issue of heterogeneity of PALs, we designed several constructs of *Oa*AEP1b‐C247A. The highly active PAL variant derived from *Oldenlandia affinis*, *Oa*AEP1b‐C247A was selected for this study due to the availability of a bacterial recombinant expression system [16, 17]. This hyperactive *Oa*AEP1b‐C247A PAL can be readily expressed in *Escherichia coli* [16, 17]. We expressed several truncated *Oa*AEP1b‐C247A proteins retaining only portions of the linker and the α6‐helix region located at the N‐terminal end of its cap domain (Fig. 1). All constructs could be overexpressed in *E. coli* as inclusion bodies. However, only *Oa*AEP1b‐C247A‐Δ351 could be readily refolded whereas all other constructs precipitated during the refolding procedure. We found that the purified protein retained a level of ligase catalytic activity comparable to the activated protein recovered after low pH treatment of the proenzyme. Thus, this work represents a cost‐effective and faster way to produce large amounts of a hyperactive ligase in *E. coli* for various attractive biotechnological and industrial applications [1].

**Fig. 1:**
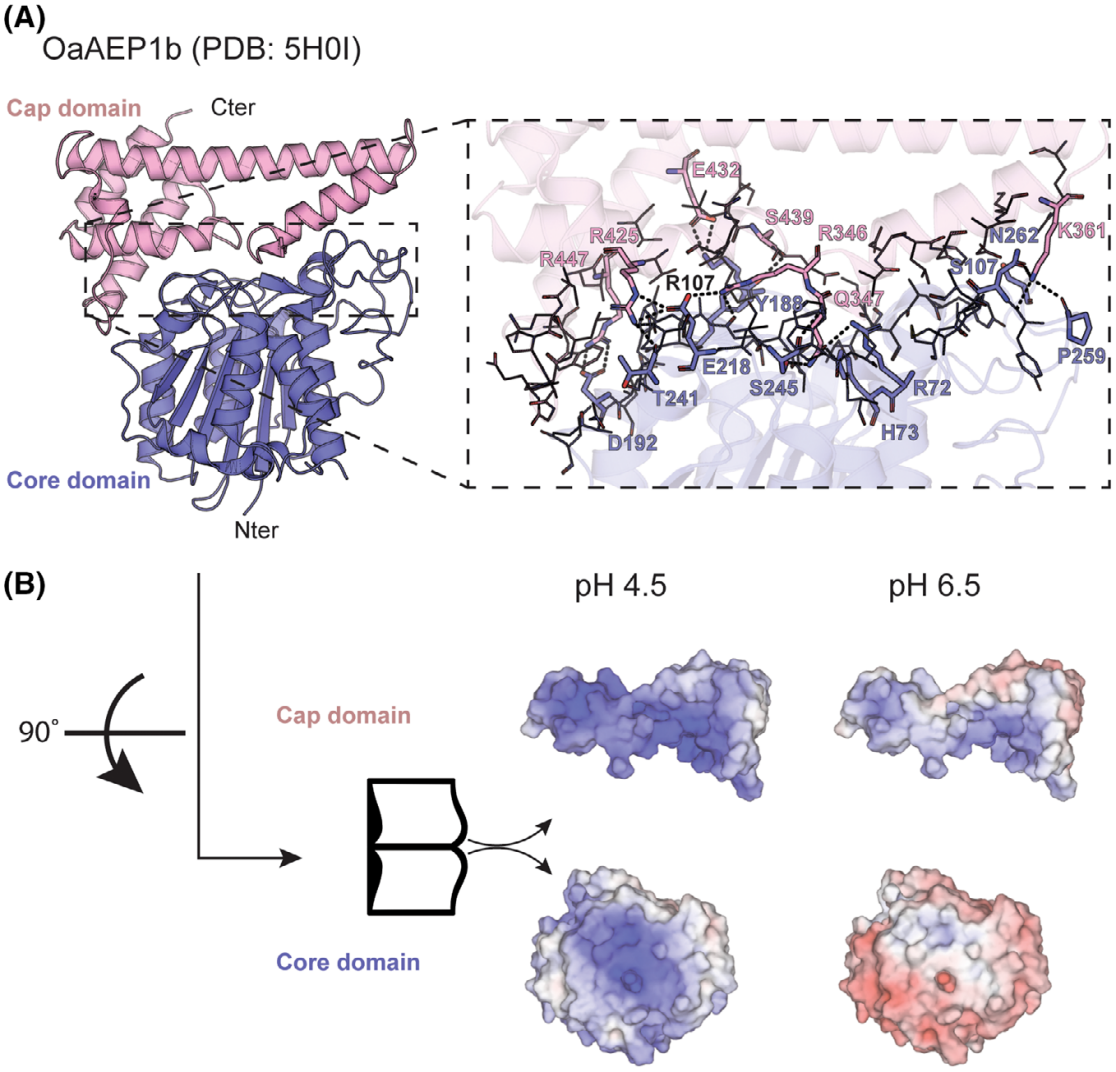
OaAEP1b core‐cap domain interface. (A) Structure of OaAEP1b in its zymogen form with residues involved in the interaction between cap and core domains represented by ball‐and‐sticks. (B) Electrostatic surface map of the core‐cap domain at pH 4.5 and 6.5, respectively, highlighting the electrostatic repulsion between both domains at pH values routinely used for acid activation of the zymogen. The electrostatic maps were calculated using the APBS server (https://server.poissonboltzmann.org) and visualized using pymol (Schrodinger Inc., New York, NY, USA).

## Results

### Analysis of the interface between the core and cap domains of OaAEP1b

The crystal structure of *Oa*AEP1b (PDB access code: 5H0I) [17] allows a precise analysis of the set of interactions established between the cap and the core domains in the zymogen form (Fig. 1A). In the context of the plant cells, the cap domain appears to regulate the activity of PALs and AEPs to prevent undesired protein processing or protein/peptide ligation. Four residues, Val344‐Val345‐Asn346‐Gln347 preceding the α6 helix (the first N‐terminal helix of the cap domain), are located at the interface between the cap and core domain. In particular, Gln347 penetrates deeply into the S1 pocket establishing several polar interactions with surrounding active site residues [17]. The interface between the cap and the core domain extends over a total surface of 1227 Å^2^ and involves 41 residues of the core domain, which make contact with 31 residues from the cap domain. A total of nine hydrogen bonds and 14 salt bridges are formed between residues from the cap and the core domain and the estimated total binding energy for this interaction is −18.8 kcal·mol^−1^ at neutral pH, as measured by Pisa (https://www.ebi.ac.uk/pdbe/pisa/). Of note, seven Glu residues are found in the interface between the cap and the core domain of *Oa*AEP1b (Fig. 1A). Separation of the two domains requires acidification of the milieu to pH values ranging between 4.0 and 4.5 with the addition of nonionic detergents such as *N*‐laurylsarcosine. At these pH values, Glu residues are no longer negatively charged, disrupting the favorable electrostatic interactions between the two domains, and favoring proteolytic cleavage *in trans* (Fig. 1B).

### Design and expression of a constitutively active OaAEP1b‐C247A

**Fig. 2:**
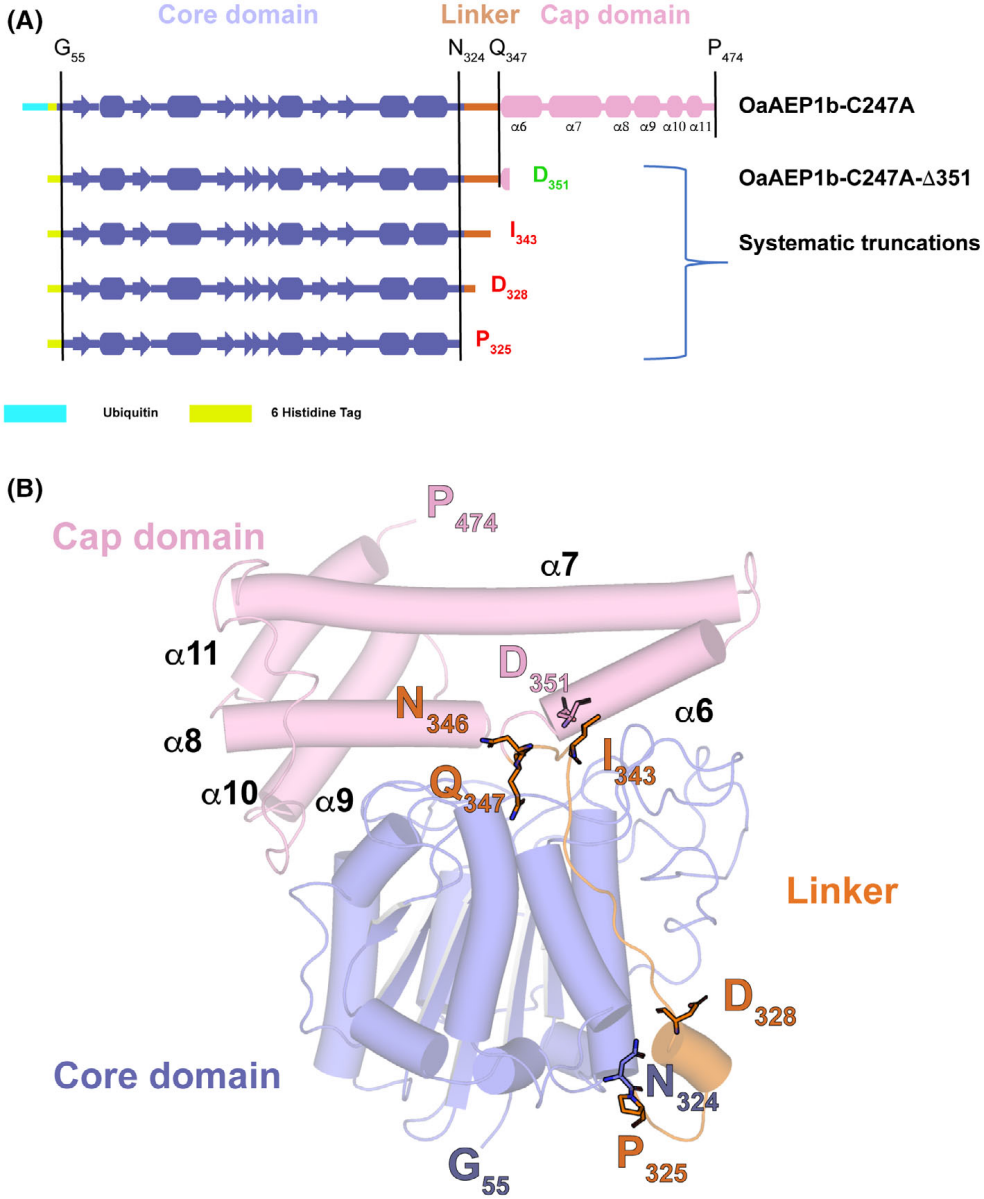
Design of truncation constructs of *Oa*AEP1b (A) Schematic view of the four constructs of *Oa*AEP1b‐C247A that were subjected to expression tests in *Escherichia coli* in this work. (B) 3D structure of *Oa*AEP1b‐C247A in its proenzyme form (PDB access code: 5H0I) (13). Residues 326–342 from the linker region are flexible and could not be traced in the electron density map. The orange dotted line represents this flexible linker region between the core and cap domains. The truncation sites introduced in our study to obtain a constitutively active peptide ligase are indicated in red.

From the analysis above on *Oa*AEP1b and from other AEPs and PAL crystal structures, it appears that the α6‐helix and the four residues Val344‐Val345‐Asn346‐Gln347 immediately preceding this α‐helix, must play an important role in stabilizing the enzyme in its zymogen form. Moreover, Asn346, Asp349, and Asp351 have been proposed to constitute possible cleavage sites leading to the mature enzyme [17]. Therefore, we designed a series of truncated constructs targeting residues located in the α6‐helix region and in the linker between the cap and core domain of *Oa*AEP1b‐C247A (Fig. 2A).

**Fig. 3:**
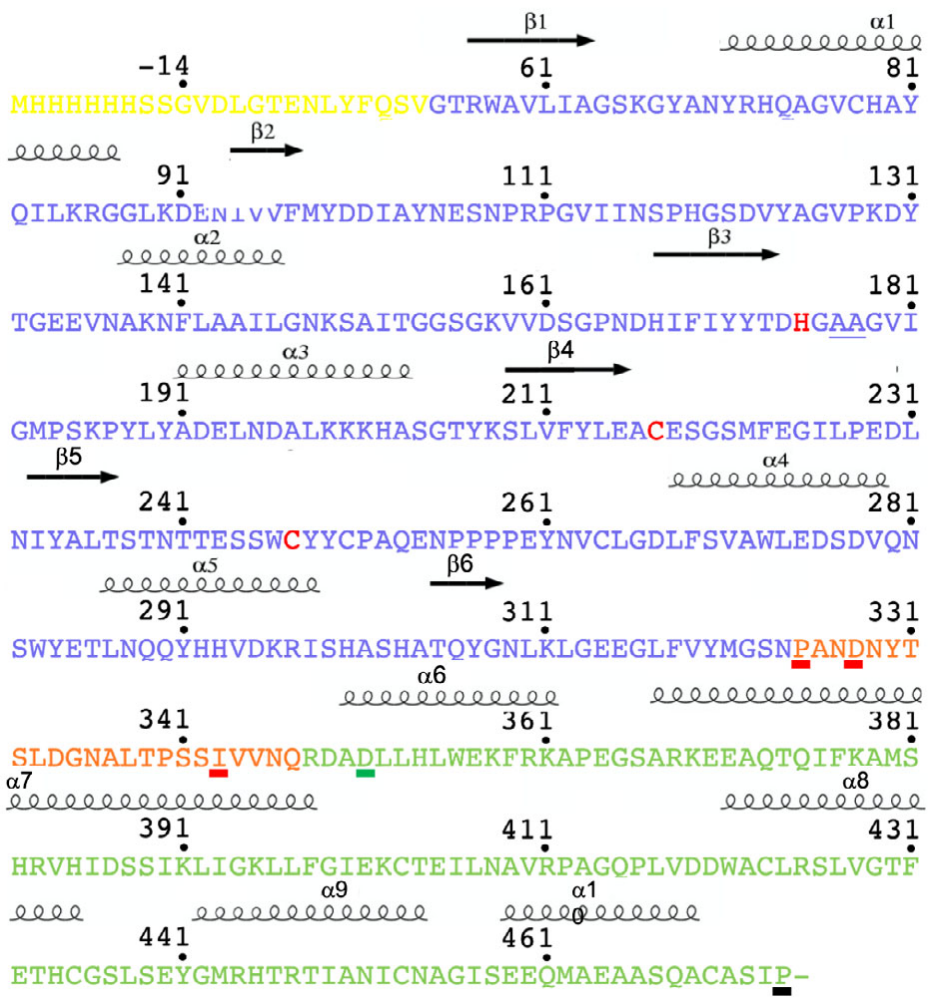
Amino acid sequence of OaAEP1b‐C247A. The amino acid sequence of the *Oa*AEP1b‐C247A proenzyme using the same color code as the 3D structure displayed in Fig. 2. The red line indicates the C‐terminus of the amino acid sequence of the respective construct (Fig. 2A). Secondary structure elements are labeled and shown above the sequence. The stretch of amino acids colored in yellow belongs to the signal peptide region, which is removed during the proenzyme maturation, with G55 becoming the N‐terminus of the mature enzyme. By contrast, the residue at the C‐terminal residue of the purified proenzyme is P474, highlighted as a black line (17). The two catalytic residues Cys217 and His175, as well as the gatekeeper residue Cys247, are highlighted in red in the amino acid sequence. Figure was created with BioRender.com.

All four constructs were expressed in *E. coli* BL21 T1R and designed to include the core domain of *Oa*AEP1b‐C247A (residues Gly55 to Asn324 according to Ref. 17 numbering) discarding the signal peptide region (residues 1–54) [17]. In addition to this core region necessary for activity, the four constructs designed included incremental sections from the linker and α6 helix encompassing putative acid‐activation sites located after Asn or Asp residues, such as Asp328 or Asn336 (Figs 2B and 3). All four constructs showed robust levels of expression in *E. coli* although the corresponding proteins were all expressed as inclusion bodies. Next, we attempted to extract proteins from the insoluble fraction by urea solubilization followed by refolding. Out of the four *Oa*AEP1b‐C247A constructs tested, only the *Oa*AEP1b‐C247A‐Δ351 protein could be refolded. For the other three truncated proteins tested, severe precipitation during the refolding procedure was observed, indicating that segments in the region spanning residues Pro325‐Asp351 are required for protein solubility.

### Refolding and purification of OaAEP1b‐C247A‐Δ351

**Fig. 4:**
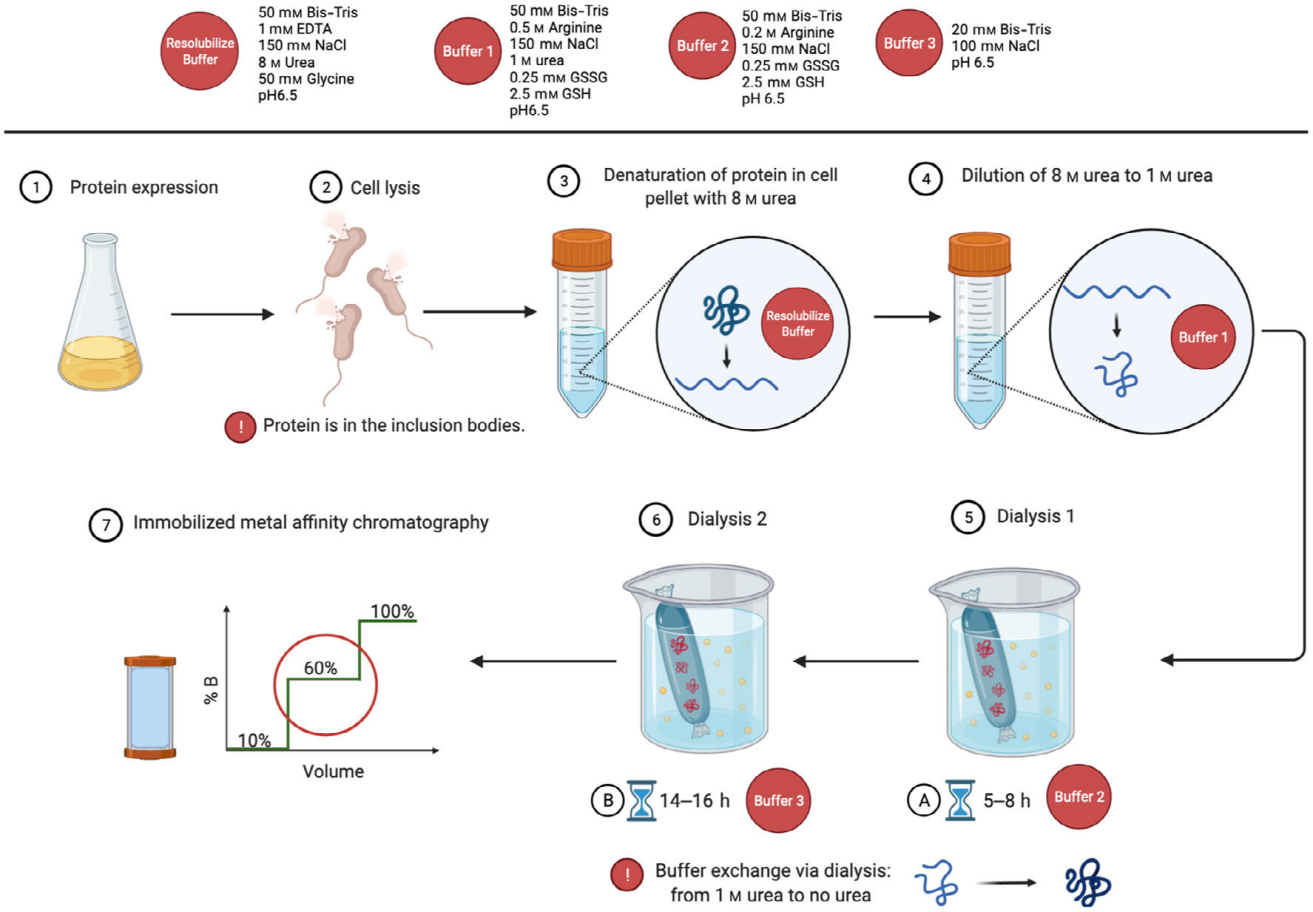
Schematic view of the dialysis refolding protocol used to purify the constitutively active *Oa*AEP1b‐C247A‐Δ351 enzyme. The target *Oa*AEP1b‐C247A‐∆351 protein was expressed as bacterial inclusion bodies and resolubilized with a buffer containing 8 m urea. Protein refolding was performed via stepwise removal of urea through dialysis against buffers 2 and 3 for 5–8 h and 14–16 h, respectively. Subsequent purification of refolded protein was done by IMAC and SEC. The composition of buffers used for purification is indicated.

**Fig. 5:**
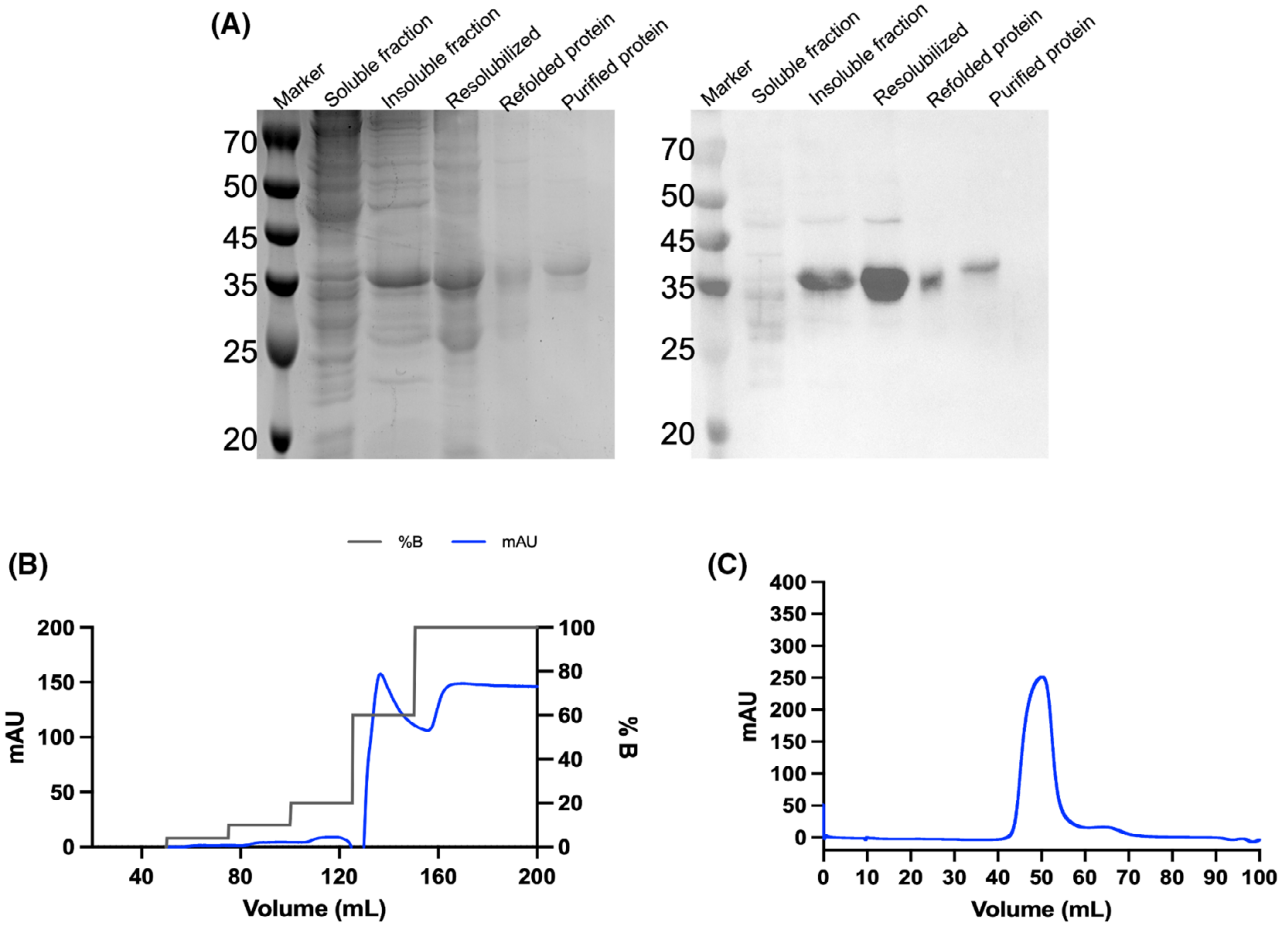
Expression in *Escherichia coli*, refolding, and purification of *Oa*AEP1b‐C247A‐Δ351. (A) The left panel shows a 12% SDS/PAGE analysis with Coomassie blue staining of the protein at various purification steps, while the right panel shows a western blot of the same gel with a commercial anti‐His antibody. A large quantity of expressed protein was observed in the insoluble fraction. The protein was resolubilized and subjected to dialysis. The refolded protein was purified using metal ion affinity chromatography followed by size exclusion chromatography to get a pure homogeneous enzyme that elutes as a monomer. (B) The metal affinity chromatogram shows the protein elution (blue curve peak at about 140 mL elution volume) following an increasing amount of imidazole buffer to the column (black lines). (C) Gel filtration of the *Oa*AEP1b‐C247A‐∆351 enzyme shows that the enzyme elutes as a monomeric species.

The expression of *Oa*AEP1b‐C247A‐∆351 was observed to be of a good level in *E. coli* inclusion bodies (Fig. 5A). Thus, the inclusion bodies were first resolubilized in 8 m urea. The protein was subsequently refolded via stepwise dilution and reduction in urea concentration from 8 to 0 m using buffer 1 and buffer 2, respectively (see Methods and Fig. 4). After the stepwise dialysis, we carried out a two‐step purification of the refolded *Oa*AEP1b‐C247A‐∆351. First, we used metal affinity chromatography (HisTrap column; Cytiva, Marlborough, MA, USA) followed by size exclusion chromatography (Superdex 200 16/600 pg; Cytiva) (Fig. 5B,C). These steps led to a pure monomeric fraction of OaAEP1b‐C247A‐∆351 (Fig. 5C). After purification, starting from a bacterial cell culture of 100 mL, we routinely obtained a yield of 1.75 mg of *Oa*AEP1b‐C247A‐∆351. Note, however, that active site titration shows that only about 53% of this protein is fully active (see below).

### Cyclization activity of OaAEP1b‐C247A‐∆351

**Fig. 6:**
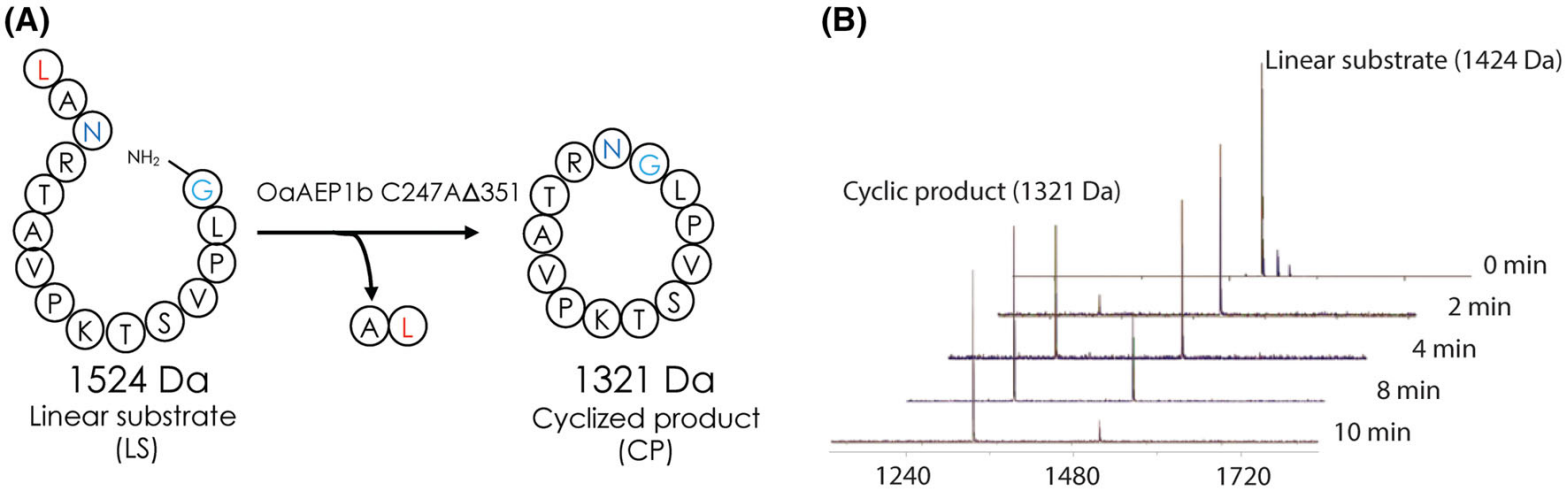
Purified *Oa*AEP1b‐C247A‐Δ351 cyclization activity assay. (A) Schematic representation of the cyclization reaction of a linear substrate (‘LS’) in the presence of the constitutively active purified PAL. (B) MALDI‐TOF mass spectra of the reaction mixture following a series of incubation time points of the linear substrate with the purified *Oa*AEP1b‐C247A‐∆351 revealing only the presence of the cyclized peptide (‘CP’) after 12 min of incubation time.

To evaluate the cyclization activity of the purified *Oa*AEP1b‐C247A‐∆351, the enzyme was tested against a linear NH2‐GLPVSTKPVATRNAL‐COOH peptide substrate (labeled ‘LS’) (Fig. 6A). The cyclization reaction was performed at 37 °C, and samples were collected every 2 min. MALDI‐TOF MS was subsequently utilized to detect a cyclized product (labeled ‘CP’). Successful cyclization carried out by the active ligase of the LS with a mass of 1524 Da would result in CP with a mass of 1321 Da (Fig. 6A). After 12 min of reaction time, *Oa*AEP1b‐C247A‐∆351 had converted the majority of the linear substrate to the circularized product. No LS peak could be detected compared with a high CP peak detected in the MALDI‐TOF mass spectra of the reaction mixture (Fig. 6B), indicating a complete cyclization reaction of the substrate by *Oa*AEP1b‐C247A‐∆351.

### Comparison of ligase activity of constitutively active vs acid‐activated PAL

**Fig. 7:**
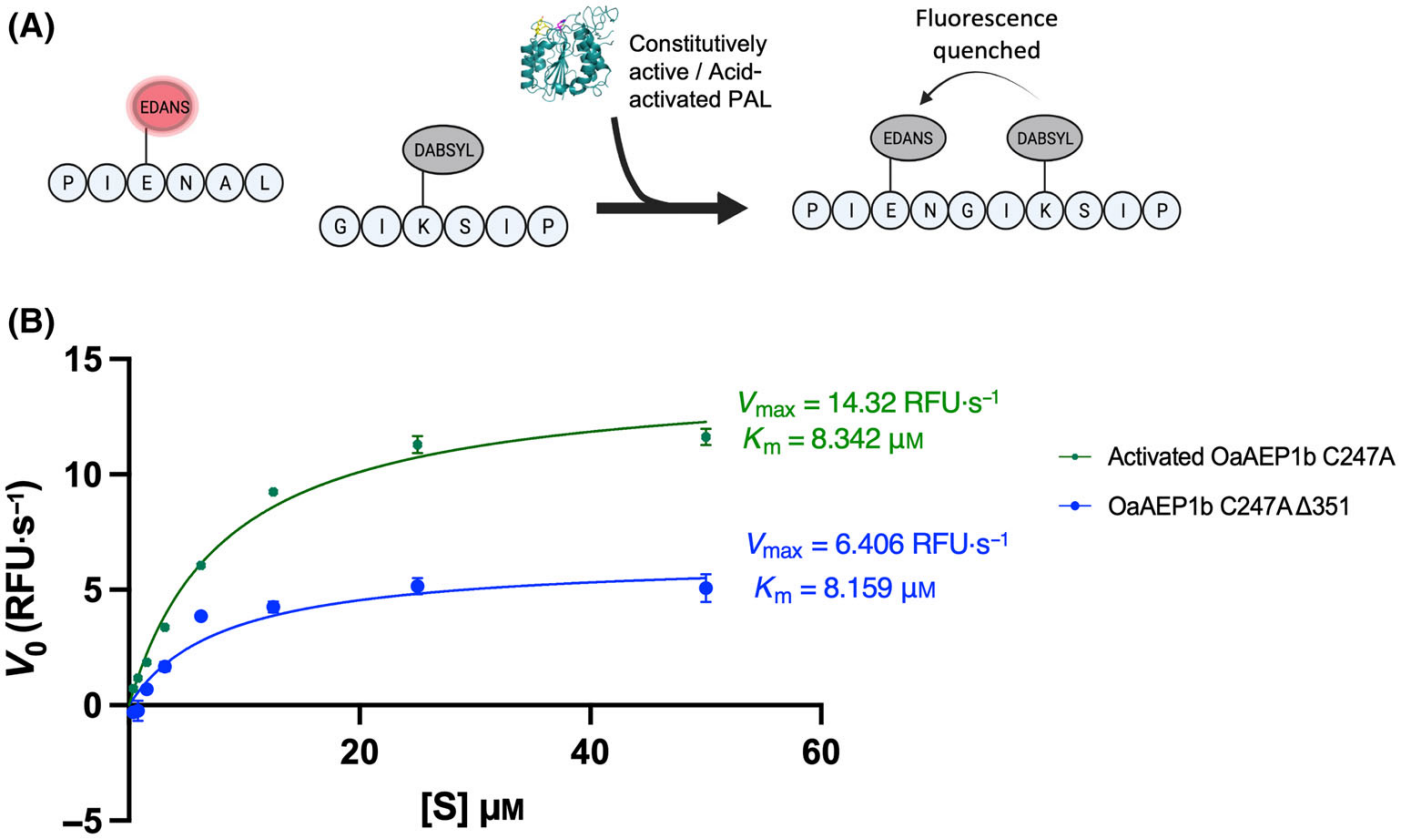
FRET ligation activity assay of *Oa*AEP1b‐C247A‐Δ351 and comparison with the acid‐activated enzyme. (A) Schematic representation of the ligation reaction of two peptides containing a FRET donor and acceptor, EDANS and DABSYL. Upon ligation, the acceptor molecule, DABSYL, comes in proximity with EDANS, resulting in a quenching of the EDANS emission (λ_em_ = 490 nm). (B) RFU values for the acid‐activated *Oa*AEP1b‐C247A enzyme (green curve) were compared with those obtained from the purified truncated *Oa*AEP1b‐C247A‐∆351 enzyme (blue curve). *V*_max_ (RFU·s^−1^) and *K*_m_ (μm) Michaelis values were deduced from two experimental repeats. The error bars represent SD from the mean.

Next, using a FRET ligation assay, we compared the ligase activity of the truncated *Oa*AEP1b‐C247A‐Δ351 with its acid‐activated zymogen counterpart. Briefly, 50 nm of either enzyme was added to a mixture of two peptides A: PIE(EDANS)YNAL and B: GIK(DABSYL)SIP. These two peptides were mixed in a A : B molar ratio of 1 : 3. Upon ligation, the fluorescence signal emission of the EDANS moiety of A (λ_em_ = 490 nm) becomes quenched by the DABSYL moiety of B (Fig. 7A). This assay allows us to follow the ligation rate between both peptides in real‐time, giving access to the kinetic parameters of the truncated enzyme. We observed that the truncated purified protein has a ligation activity comparable (about 2‐fold less) to its acid‐activated zymogen counterpart and previously reported *Oa*AEP1b‐C247A [17]. The *V*_max_ and *K*_m_ values are 6.40 RFU·s^−1^ and 8.16 μm, respectively, for *Oa*AEP1b‐C247A‐∆351 compared with 14.32 RFU·s^−1^ and 8.34 μm for acid‐activated *Oa*AEP1b‐C247A *V*_max_ and *K*_m_ values, respectively (Fig. 7B). As the constitutively active PAL was obtained using a refolding protocol, the exact final proportion of *Oa*AEP1b‐C247A‐Δ351 proteins adopting an active conformation is not known, giving some uncertainty on the determination of the kinetic parameters. Thus, in order to refine the comparison of the activity of the refolded enzyme with the acid‐activated one, we performed the titration of their active sites following the procedure outlined in Ref. [32].

**Fig. 8:**
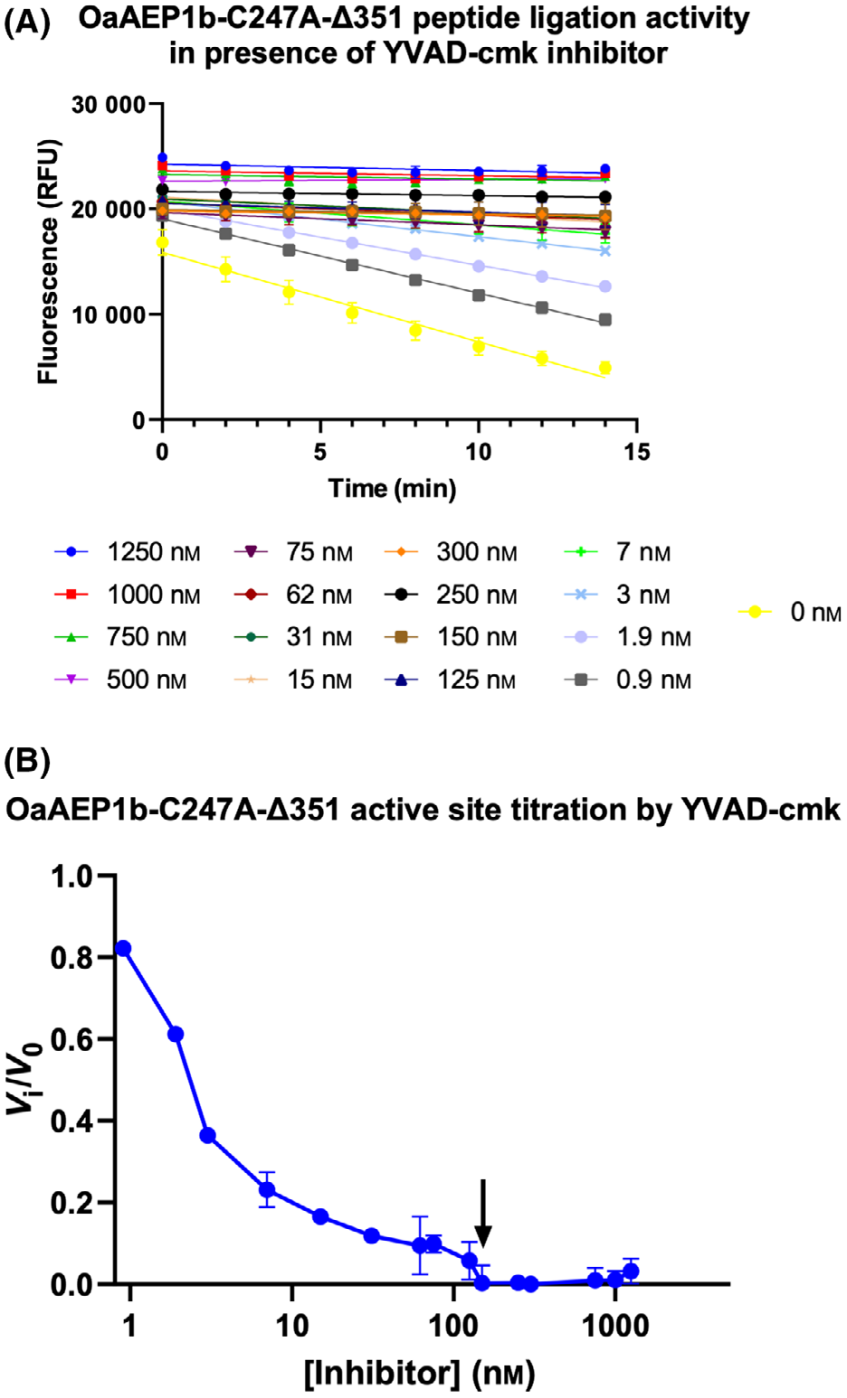
Active site titration of *Oa*AEP1b‐C247A‐Δ351. (A) *Oa*AEP1b‐C247A‐Δ351 activity was measured by FRET ligation assay in the presence of increasing concentrations of ac‐YVAD‐cmk covalent inhibitor [19]. The measured concentration of the enzyme is 280 nm (B) Slopes from linear regression at various inhibitor concentrations. Individual curves were plotted as a fraction of the slope obtained in the absence of an inhibitor. The arrow indicates the minimal concentration of inhibitor necessary for complete inhibition of the ligation activity (150 nm). The error bars represent SD from the mean.

To understand the difference in the *V*_max_ between *Oa*AEP1b‐C247A‐∆351 and acid‐activated *Oa*AEP1b‐C247A we performed an active site titration of *Oa*AEP1b‐C247A‐∆351 using a FRET ligation assay, after a 1 h incubation with varying concentrations of a covalent AEP inhibitor, Ac‐YVAD‐cmk [19]. The result of the active site titration showed that about 53% of the measured protein concentration is active and amenable to complete inhibition (Fig. 8). Remarkably, this difference in the concentration of active protein matches the measured difference in *V*_max_ and suggests that the activity of the *Oa*AEP1b‐C247A‐∆351 is very similar to the activity of the acid‐activated *Oa*AEP1b‐C247A.

### Conjugation of the tRNA methyltransferase, TrmJ with a fluorescent peptide

**Fig. 9:**
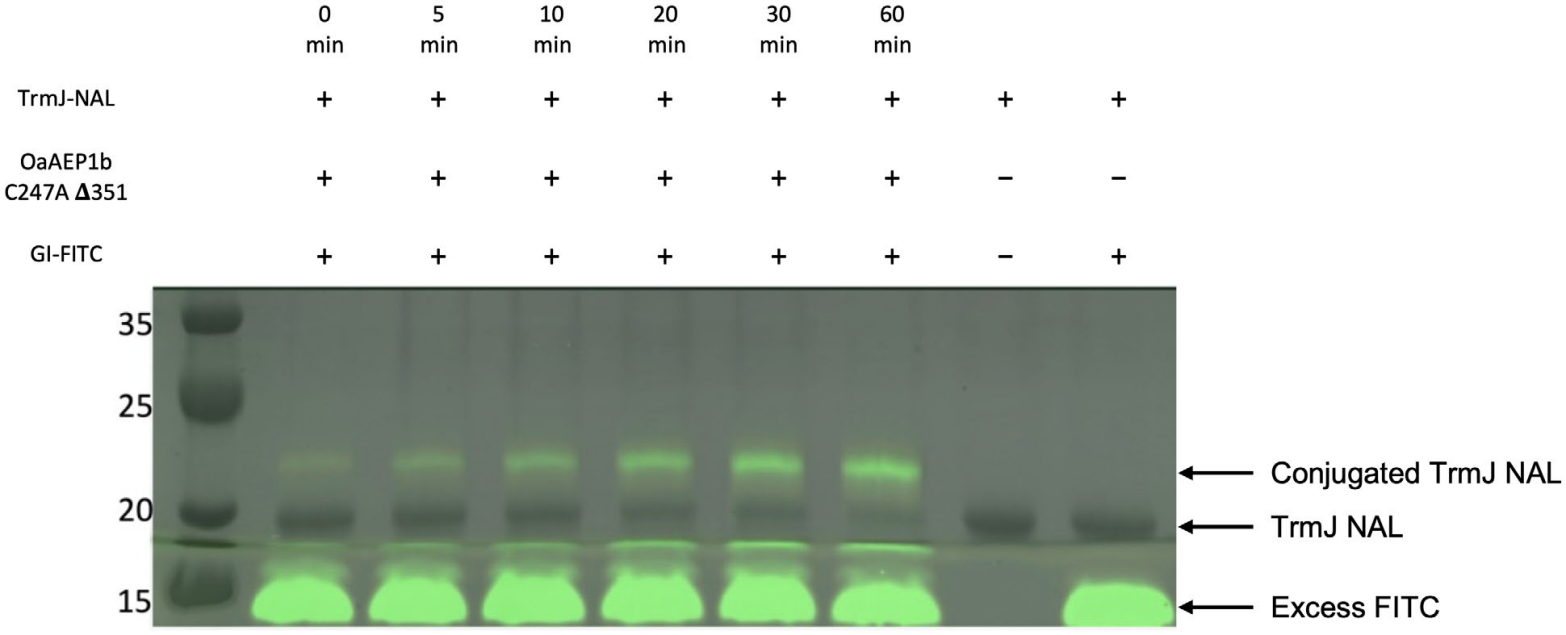
Conjugation of a tRNA methyltransferase, TrmJ using *Oa*AEP1b‐C247A‐Δ351. SDS/PAGE analysis of protein TrmJ‐NAL conjugation with a short fluorescence peptide (GI‐FITC). The reaction was carried out for an hour and sampled every 5, 10, and 30 min showing the time‐dependent formation of the fluorescent conjugate of TrmJ.

To evaluate *Oa*AEP1b‐C247A ∆351 conjugation capability, we conjugated a protein of 20 kDa, tRNA methyltransferase, TrmJ [33]. We modified TrmJ to include the C‐terminal *Oa*AEP1b‐C247A ∆351 preferred tripeptide recognition motif (Asn‐Ala‐Leu). Using 200 nm of *Oa*AEP1b‐C247A ∆351, we were able to conjugate TrmJ present in the solution with a short fluorescence peptide consisting of an N‐terminal Gly/Ile (GIGGIYRK‐FITC). The conjugation rate at 37 °C was analyzed using SDS/PAGE at six different time points. An increment of the FITC signal was observed at every time point, and after an hour of reaction time, most of TrmJ was labeled with FITC (Fig. 9). These results demonstrated that the constitutively active *Oa*AEP1b‐C247A ∆351 can efficiently conjugate a protein.

## Discussion

The highly active PAL single mutant *Oa*AEP1b‐C247A was selected for this study due to the availability of a convenient bacterial recombinant expression system, while other hyperactive PALs require expression in insect cell systems [15, 16, 17]. Here, we showed that the bacterial recombinant expression of a constitutively active PAL is possible by introducing systematic truncations along the ⍺6 helix, which penetrates into the enzyme active site. We found that these truncations resulted in the protein being expressed as inclusion bodies, demonstrating that the cap domain provides a set of polar interactions with the core domain that are essential for soluble recombinant expression of the proenzyme. Moreover, constructs entirely devoid of the ⍺6‐helix displayed severe precipitation during the purification process. By contrast, construct *Oa*AEP1b‐C247A‐∆351, which retains a small portion of ⍺6‐helix enabled the purification of the protein from inclusion bodies without any severe precipitation. This result suggests that the presence of a portion of the ⍺6‐helix is crucial in maintaining protein stability in solution. In summary, these results indicate that it is possible to express the *Oa*AEP1b‐C247A enzyme devoid of its inhibitory cap domain while retaining a level of catalytic activity similar to the acid‐activated species.

An important question is whether the *Oa*AEP1b‐C247A‐∆351 construct derived in this work gives an economical advantage compared with the original construct that needs acid activation to obtain an enzymatically competent form [16, 17]. In our hands, using the *Oa*AEP1b C247A proenzyme as starting material [17], the final yield after acid activation and purification is about 1–2 mg·L^−1^ of LB culture, which is significantly lower than what we obtain in the present work with a yield of refolded and active OaAEP1b‐C247A‐∆351 enzyme of ~ 0.9 mg/100 mL.

Despite having retained a very small portion of the cap domain in our design, *Oa*AEP1b‐C247A‐∆351 retains high enzymatic activity in an intramolecular cyclization assay. We were able to detect the complete conversion of the linear substrate to the cyclized product (Fig. 6). Likewise, taking into account the number of active enzymes, in an intermolecular ligation assay, the catalytic rate observed for the refolded *Oa*AEP1b‐C247A‐∆351 was comparable with its acid‐activated counterpart. Nonetheless, the intermolecular ligation of two peptides and the conjugation assays were slower than intramolecular cyclization. An intramolecular cyclization reaction generally proceeds faster due to the incoming nucleophile being present in cis within the peptide substrate. By contrast, for intermolecular ligation, a molar excess of electrophilic and nucleophilic substrate peptides is required for efficient catalysis of the reaction [10].

It is noteworthy that a bacterial recombinant expression of a truncated *Oa*AEP1b‐C247A was published during the course of the present work [34]. In this design, the truncated enzyme comprised residue D328 and was completely devoid of the ⍺6‐helix. The truncated *Oa*AEP1b‐C247A protein was reported to have comparable catalytic kinetics to its acid‐activated counterpart [34]. However, the reported yield was much lower as compared to the yield obtained in the present work, which could be due to the complete removal of the ⍺6‐helix, resulting in a less stable enzyme during the expression and purification steps.

## Conclusion

So far, enzymatically active forms of either PALs or AEPs were only obtained via activation under acidic conditions [15, 16, 17]. This step leads to the introduction of a heterogeneous population of enzymes due to the multiple accessible activation sites present in the proenzyme, thus, limiting the quality and quantity of homogenous active PALs obtained.

We carried out systematic truncations of the *Oa*AEP1b‐C274A proenzyme to address this issue. As a result, we identified *Oa*AEP1b‐C247A‐∆351, which showed both good expression levels and activity in a bacterial expression system. The expression and purification of a constitutively active PAL alleviate the need for a tedious activation step and additional purification procedures. This expression and purification protocol leads to an enzyme endowed with comparable ligation kinetics as its acid‐activated counterparts. Remarkably, compared with currently available acid‐activation methods for PAL expression and purification, the yield of active ligase is increased from 1 to 2 mg·L^−1^ of *E. coli* culture to more than 9 mg·L^−1^ for the current method. As a cautionary note, scaling up in the laboratory does not necessarily translate into an exact tenfold increase in yield, as large volumes of refolding buffers would have to be handled when using several liters of cell culture.

We foresee that the first successful purification of a stable and constitutively active ligase in a bacterial expression system described here could constitute a cost‐effective way for the large‐scale production of several hyperactive ligases. In turn, these constitutively active enzymes will be convenient tools for various attractive industrial applications that require protein conjugation such as for the manufacturing of antibody‐drug conjugates.

## Methods

### Design and expression of constitutively active *Oa*AEP1b‐C247A‐Δ351

The expression constructs spanning residues Gly55 to Asp351, with an N‐terminal hexahistidine tag followed by a TEV cleavage site, were synthesized by BioBasic (Singapore City, Singapore). These constructs were expressed in *E. coli* BL21 (T1R) cells and cultivated at 37 °C to an OD_600_ ~ 1 in LB media (Biobasic). The proteins were overexpressed following induction with 0.5 mm IPTG at 18 °C for 18 h. Cells were pelleted and stored at −80 °C before purification.

### Refolding and purification of constitutively active *Oa*AEP1b‐C247A‐Δ351 using dialysis method

With all steps performed at 4 °C, protein purification was achieved by resuspending thawed pellets in 30 mL of lysis buffer (100 mm Bis‐Tris, pH 6.5, 500 mm NaCl, 10% (v/v) glycerol), sonicating the pellets followed by clearing the lysates by centrifugation at 58 000 ***g*** for 45 min. The insoluble fraction was resolubilized in 10 mL of resolubilizing buffer (50 mm Bis‐Tris, pH 6.5, 150 mm NaCl, 1 mm EDTA, 50 mm Glycine, 8 m urea) overnight, with agitation. The concentration of denatured protein was determined using nanodrop and diluted to ~ 2 mg mL^−1^ with buffer 1 (50 mm Bis‐Tris, pH 6.5, 150 mm NaCl, 500 mm l‐Arginine, 1 m urea, 0.25 mm l‐glutathione oxidized, 2.5 mm l‐glutathione reduced). The diluted denatured protein was dialyzed against buffer 2 (50 mm Bis‐Tris, pH 6.5, 150 mm NaCl, 200 mm l‐arginine, 0.125 mm l‐glutathione oxidized, 1.25 mm l‐glutathione reduced) for 8 h, followed by two buffer exchanges with buffer 3 (20 mm Bis‐Tris, pH 6.5, 150 mm NaCl), for a duration of 8 h for each buffer exchange. The refolded proteins were purified by affinity chromatography (HisTrap column; Cytiva), followed by size exclusion chromatography (HiLoad 16/600 Superdex 200; Cytiva). The proteins eluted as monomers during size exclusion purification. *Oa*AEP1b‐C247A‐Δ351 was concentrated to 1 mg·mL^−1^ in 20 mm Bis‐Tris, pH 6.5, 150 mm NaCl, 5% (v/v) glycerol using centrifugation and concentrators with a 10 kDa cut‐off (Amicon; Merck KGaA, Darmstadt, Germany). Aliquots were flash‐frozen in liquid nitrogen and stored at −80 °C until use.

Protein samples were collected after resolubilization, dialysis, and purification and were analyzed with SDS/PAGE. Western blot analysis was also carried out using anti‐His antibody obtained from Sigma Aldrich (Saint Louis, MO, USA) (catalog number: SAB4301134) to validate the purification of the protein. The purified protein was trypsin digested using a standard protocol and the digested fragments were analyzed via mass spectrometry (Table S1).

### Purification and expression of full‐length *Oa*AEP1b‐C247A

The full‐length *Oa*AEP1b‐C247A construct was synthesized by BioBasic and was expressed in *E. coli* BL21 (T1R) cells. Expression and activation of *Oa*AEP1b‐C247A were done according to reference [17].

### Peptide cyclization assay

The peptide used for cyclization assay was purchased commercially from Genscript Biotech (Kallang, Singapore), NH_2_‐GLPVSTKPVATRNAL‐COOH. Cyclization assays were performed in 50 μL reaction mixtures containing 20 mm phosphate buffer, pH 6.5, ligases (40 nm), and peptide substrates (20 μm). Reaction was performed at 37 °C, for 1 h. The cyclization product was analyzed by MALDI‐TOF MS (ABI 4800 MALDI‐TOF/TOF; Applied Biosystems, Waltham, MA, USA).

### Kinetics assay

The kinetic properties of the peptide ligation of the constitutively active PAL were studied using a FRET assay. Two peptides synthesized by Genscript Biotech: PIE{EDANS}YNAL and GIK{DABSYL}SIP were mixed at a molar ratio of 1 : 3. Upon ligation, the peptide PIE{EDANS}YNGIK{DABSYL}SIP is produced. Fifty nanomolar of PAL enzyme is mixed with various concentrations of the peptide mixture. The EDANS fluorescence signal was measured with an excitation wavelength of 336 nm and an emission wavelength of 490 nm. A reduction in EDANS fluorescence signal occurs upon ligation of the two peptides due to quenching by DABSYL. The variation in fluorescence signal for each substrate mixture concentration was measured after the addition of the enzyme to initiate the reaction. The rate of decrease in fluorescence signal during the first 30 s after enzyme addition was plotted against the substrate concentration to obtain the *V*_max_, *k*_cat_, and *K*_m_ values for each enzyme.

### Active site titration

We followed the procedure described in reference [32]. The enzyme preparation was diluted to a concentration of 280 nm using a buffer containing 20 mm sodium phosphate at pH 6.5 and 5 mm 2‐mercaptoethanol. Solutions containing serial twofold dilution of inhibitor YVAD‐cmk [19] were prepared in a black microtiter plate (Greiner Bio‐One GmbH, Kremsmünster, Austria) using buffer as diluent. The enzyme was subsequently added to the wells containing the inhibitor to a final volume of 50 μL. The plate was incubated for 1 h at room temperature before adding FRET peptides (PIE{EDANS}YNAL and GIK{DABSYL}SIP), which were mixed at a molar ratio of 1 : 3 giving a final enzyme: substrate molar ratio of 1 : 200. The EDANS fluorescence signal was measured with an excitation wavelength of 336 nm and an emission wavelength of 490 nm. Relative fluorescence units (RFU) of quenched EDANS signal were plotted against time. The value of the initial velocity (*V*_i_) was determined from the slope of the RFU(t) curve. The measured value of *V*_i_ was subsequently normalized by dividing with the initial rate obtained in the absence of inhibitor (control V_0_). The calculated *V*_i_/*V*_0_ ratio was plotted against inhibitor concentrations, generating an inhibition curve. The titer of the enzyme active site was then inferred from the intercept of this inhibition curve with the *x*‐axis, assuming a 1 : 1 interaction between enzyme and inhibitor, which is in agreement with experimental crystallographic structures of homologous PALs with a peptide substrate published previously [19, 35].

### Conjugation of TrmJ

A concentration of 200 nm of *Oa*AEP1b‐C247A‐∆351 was used to conjugate 10 μm of TrmJ‐NAL [33] with 50 μm of a short fluorescence peptide synthesized by Genscript Biotech: GIGGIYRK‐FITC. This reaction was carried out in a 20 mm NaH_2_PO_4_, pH 6.5 at 37 °C for 1 h with a final volume of 500 μL. A volume of 50 μL of the reaction was mixed with 5 × SDS loading dye after 5, 10, 20, 30, and 60 min. The amount of conjugated TrmJ‐NAL at all time points was then analyzed using SDS/PAGE.

## Conflict of interest

JL and CFL are co‐founders and shareholders of Singzyme Pte Ltd, which has acquired licenses on peptide ligase technology.

## Author contributions

YHW designed the experiments. YHW and NC planned and performed the experiments. YHW, NC, AES, CFL, and JL analyzed the data and wrote the paper.

## Supporting information


**Table S1.** MASCOT sequence query results of purified *Oa*AEP1b‐C247A Δ351.
**Data S1.** OaAEP1b‐C247A‐∆351 amino‐acid sequence.

## Data Availability

This manuscript has Supporting Information available online. Materials reported in this manuscript are available from the authors upon reasonable request, after M.T.A. has been signed.

## References

[1] Bagert JD and Muir TW (2021) Molecular epigenetics: chemical biology tools come of age. Annu Rev Biochem 90, 287–320. 34153213 10.1146/annurev-biochem-080120-021109 PMC8284505

[2] Schmidt M , Toplak A , Quaedflieg PJ and Nuijens T (2017) Enzyme‐mediated ligation technologies for peptides and proteins. Curr Opin Chem Biol 38, 1–7. 28229906 10.1016/j.cbpa.2017.01.017

[3] Cao Y , Nguyen GKT , Tam JP and Liu C‐F (2015) Butelase‐mediated synthesis of protein thioesters and its application for tandem chemoenzymatic ligation. Chem Commun 51, 17289–17292. 10.1039/c5cc07227a 26462854

[4] Bi X , Yin J , Nguyen GKT , Rao C , Halim NBA , Hemu X , Tam JP and Liu CF (2017) Enzymatic engineering of live bacterial cell surfaces using butelase 1. Angew Chem Int Ed Engl 56, 7822–7825. 28524544 10.1002/anie.201703317

[5] Kwon S , Duarte JN , Li Z , Ling JJ , Cheneval O , Durek T , Schroeder CI , Craik DJ and Ploegh HL (2018) Targeted delivery of cyclotides via conjugation to a nanobody. ACS Chem Biol 13, 2973–2980. 30248263 10.1021/acschembio.8b00653

[6] Cao Y , Nguyen GKT , Chuah S , Tam JP and Liu C‐F (2016) Butelase‐mediated ligation as an efficient bioconjugation method for the synthesis of peptide dendrimers. Bioconjug Chem 27, 2592–2596. 27723303 10.1021/acs.bioconjchem.6b00538

[7] Nguyen GKT , Cao Y , Wang W , Liu CF and Tam JP (2015) Site‐specific N‐terminal labeling of peptides and proteins using butelase 1 and thiodepsipeptide. Angew Chem Int Ed Engl 54, 15694–15698. 26563575 10.1002/anie.201506810

[8] Nguyen GKT , Hemu X , Quek JP and Tam JP (2016) Butelase‐mediated macrocyclization of d‐amino‐acid‐containing peptides. Angew Chem Int Ed Engl 55, 12802–12806. 27624217 10.1002/anie.201607188

[9] Nguyen GKT , Kam A , Loo S , Jansson AE , Pan LX and Tam JP (2015) Butelase 1: a versatile ligase for peptide and protein macrocyclization. J Am Chem Soc 137, 15398–15401. 26633100 10.1021/jacs.5b11014

[10] Cao Y , Nguyen GKT , Qiu Y , Liu C‐F , Tam JP and Hemu X (2016) Butelase‐mediated cyclization and ligation of peptides and proteins. Nat Protoc 11, 1977–1988. 27658013 10.1038/nprot.2016.118

[11] Nguyen GKT , Wang S , Qiu Y , Hemu X , Lian Y and Tam JP (2014) Butelase 1 is an Asx‐specific ligase enabling peptide macrocyclization and synthesis. Nat Chem Biol 10, 732–738. 25038786 10.1038/nchembio.1586

[12] Mao H , Hart SA , Schink A and Pollok BA (2004) Sortase‐mediated protein ligation: a new method for protein engineering. J Am Chem Soc 126, 2670–2671. 14995162 10.1021/ja039915e

[13] Jackson MA , Nguyen LTT , Gilding EK , Durek T and Craik DJ (2020) Make it or break it: plant AEPs on stage in biotechnology. Biotechnol Adv 45, 107651. 33141031 10.1016/j.biotechadv.2020.107651

[14] James AM , Haywood J and Mylne JS (2018) Macrocyclization by asparaginyl endopeptidases. New Phytol 218, 923–928. 28322452 10.1111/nph.14511

[15] Hemu X , El Sahili A , Hu S , Wong K , Chen Y , Wong YH , Zhang X , Serra A , Goh BC , Darwis DA *et al*. (2019) Structural determinants for peptide‐bond formation by asparaginyl ligases. Proc Natl Acad Sci USA 116, 11737–11746. 31123145 10.1073/pnas.1818568116 PMC6576118

[16] Harris KS , Durek T , Kaas Q , Poth AG , Gilding EK , Conlan BF , Saska I , Daly NL , Van Der Weerden NL , Craik DJ *et al*. (2015) Efficient backbone cyclization of linear peptides by a recombinant asparaginyl endopeptidase. Nat Commun 6, 10199. 26680698 10.1038/ncomms10199 PMC4703859

[17] Yang R , Wong YH , Nguyen GKTT , Tam JP , Lescar J and Wu B (2017) Engineering a catalytically efficient recombinant protein ligase. J Am Chem Soc 139, 5351–5358. 28199119 10.1021/jacs.6b12637

[18] Dall E and Brandstetter H (2013) Mechanistic and structural studies on legumain explain its zymogenicity, distinct activation pathways, and regulation. Proc Natl Acad Sci USA 110, 10940–10945. 23776206 10.1073/pnas.1300686110 PMC3703970

[19] Dall E , Zauner FB , Soh WT , Demir F , Dahms SO , Cabrele C , Huesgen PF and Brandstetter H (2020) Structural and functional studies of *Arabidopsis thaliana* legumain beta reveal isoform specific mechanisms of activation and substrate recognition. J Biol Chem 295, 13047–13064. 32719006 10.1074/jbc.RA120.014478 PMC7489914

[20] Bernath‐Levin K , Nelson C , Elliott AG , Jayasena AS , Millar AH , Craik DJ and Mylne JS (2015) Peptide macrocyclization by a bifunctional endoprotease. Chem Biol 22, 571–582. 25960260 10.1016/j.chembiol.2015.04.010

[21] Zauner FB , Elsässer B , Dall E , Cabrele C and Brandstetter H (2018) Structural analyses of *Arabidopsis thaliana* legumain reveal differential recognition and processing of proteolysis and ligation substrates. J Biol Chem 293, 8934–8946. 29628443 10.1074/jbc.M117.817031 PMC5995516

[22] Zauner FB , Dall E , Regl C , Grassi L , Huber CG , Cabrele C and Brandstetter H (2018) Crystal structure of plant legumain reveals a unique two‐chain state with pH‐dependent activity regulation. Plant Cell 30, 686–699. 29453229 10.1105/tpc.17.00963 PMC5894848

[23] James AM , Haywood J , Leroux J , Ignasiak K , Elliott AG , Schmidberger JW , Fisher MF , Nonis SG , Fenske R , Bond CS *et al*. (2019) The macrocyclizing protease butelase 1 remains autocatalytic and reveals the structural basis for ligase activity. Plant J 98, 988–999. 30790358 10.1111/tpj.14293

[24] Hemu X , El Sahili A , Hu S , Zhang X , Serra A , Goh BC , Darwis DA , Chen MW , Sze SK , Liu C *et al*. (2020) Turning an asparaginyl endopeptidase into a peptide ligase. ACS Catal 10, 8825–8834.

[25] Dall E , Stanojlovic V , Demir F , Briza P , Dahms SO , Huesgen PF , Cabrele C and Brandstetter H (2021) The peptide ligase activity of human legumain depends on fold stabilization and balanced substrate affinities. ACS Catal 11, 11885–11896. 34621593 10.1021/acscatal.1c02057 PMC8491156

[26] Haywood J , Schmidberger JW , James AM , Nonis SG , Sukhoverkov KV , Elias M , Bond CS and Mylne JS (2018) Structural basis of ribosomal peptide macrocyclization in plants. Elife 7, e32955. 29384475 10.7554/eLife.32955 PMC5834244

[27] Zhao L , Hua T , Crowley C , Ru H , Ni X , Shaw N , Jiao L , Ding W , Qu L , Hung LW *et al*. (2014) Structural analysis of asparaginyl endopeptidase reveals the activation mechanism and a reversible intermediate maturation stage. Cell Res 24, 344–358. 24407422 10.1038/cr.2014.4 PMC3945893

[28] Jackson MA , Gilding EK , Shafee T , Harris KS , Kaas Q , Poon S , Yap K , Jia H , Guarino R , Chan LY *et al*. (2018) Molecular basis for the production of cyclic peptides by plant asparaginyl endopeptidases. Nat Commun 9, 1–12. 29925835 10.1038/s41467-018-04669-9 PMC6010433

[29] Kuroyanagi M , Nishimura M and Hara‐Nishimura I (2002) Activation of Arabidopsis vacuolar processing enzyme by self‐catalytic removal of an auto‐inhibitory domain of the C‐terminal propeptide. Plant Cell Physiol 43, 143–151. 11867693 10.1093/pcp/pcf035

[30] Mulvenna JP , Mylne JS , Bharathi R , Burton RA , Shirley NJ , Fincher GB , Anderson MA and Craik DJ (2006) Discovery of cyclotide‐like protein sequences in graminaceous crop plants: ancestral precursors of circular proteins? Plant Cell 18, 2134–2144. 16935986 10.1105/tpc.106.042812 PMC1560918

[31] Mylne JS , Chan LY , Chanson AH , Daly NL , Schaefer H , Bailey TL , Nguyencong P , Cascales L and Craik DJ (2012) Cyclic peptides arising by evolutionary parallelism via asparaginyl‐endopeptidase‐mediated biosynthesis. Plant Cell 24, 2765–2778. 22822203 10.1105/tpc.112.099085 PMC3426113

[32] Harris KS , Guarino RF , Dissanayake RS , Quimbar P , McCorkelle OC , Poon S , Kaas Q , Durek T , Gilding EK , Jackson MA *et al*. (2019) A suite of kinetically superior AEP ligases can cyclise an intrinsically disordered protein. Sci Rep 9, 1–13. 31346249 10.1038/s41598-019-47273-7 PMC6658665

[33] Jaroensuk J , Atichartpongkul S , Chionh YH , Hwa Wong Y , Liew CW , McBee ME , Thongdee N , Prestwich EG , DeMott MS , Mongkolsuk S *et al*. (2016) Methylation at position 32 of tRNA catalyzed by TrmJ alters oxidative stress response in *Pseudomonas aeruginosa* . Nucleic Acids Res 44, 10834–10848. 27683218 10.1093/nar/gkw870 PMC5159551

[34] Tang TMS , Cardella D , Lander AJ , Li X , Escudero JS , Tsai YH and Luk LYP (2020) Use of an asparaginyl endopeptidase for chemo‐enzymatic peptide and protein labeling. Chem Sci 11, 5881–5888. 32874509 10.1039/d0sc02023k PMC7441500

[35] Hu S , El Sahili A , Kishore S , Wong YH , Hemu X , Goh BC , Wang Z , Tam JP , Liu C‐F and Lescar J (2022) Structural basis for proenzyme maturation, substrate recognition and ligation by a hyperactive peptide asparaginyl ligase. Plant Cell 34, 4936–4949. 36099055 10.1093/plcell/koac281 PMC9709980

