# Supplementary material for: On the design of a constitutively active peptide asparaginyl ligase for facile protein conjugation
Source: FEBS Open Bio. 2023 Mar 28;13(6):1095–106. doi: 10.1002/2211-5463.13575 (PMC10240336; doi:10.1002/2211-5463.13575)
Supplement: Supplementary file 1 — Table S1. MASCOT sequence query results of purified OaAEP1b‐C247A Δ351. Data S1. OaAEP1b‐C247A‐∆351 amino‐acid sequence. [file FEB4-13-1095-s001.docx]

**On the design of a constitutively active peptide asparaginyl ligase for facile protein conjugation**

Niying Chua ^1,2, *^, Yee Hwa Wong^1,2*^ , Abbas El Sahili^1,2^, Chuan Fa Liu^1^ & Julien Lescar^1,2,#^

^1^School of Biological Sciences, Nanyang Technological University. 60 Nanyang Drive, Singapore 637551

^2^NTU Institute of Structural Biology, Experimental Medicine Building, 59 Nanyang Drive, Singapore 636921

**Supporting information**

**Contains *Oa*AEP1b-C247A-**Δ**351 amino-acid sequence and one supplementary Table.**

***Oa*AEP1b-C247A-**Δ**351 amino-acid sequence**

MHHHHHHSSGVDLGTENLYFQSVGTRWAVLIAGSKGYANYRHQAGVCHAYQILKRGGLKDENIVVFMYDDIAYNESNPRPGVIINSPHGSDVYAGVPKDYTGEEVNAKNFLAAILGNKSAITGGSGKVVDSGPNDHIFIYYTDHGAAGVIGMPSKPYLYADELNDALKKKHASGTYKSLVFYLEACESGSMFEGILPEDLNIYALTSTNTTESSWCYYCPAQENPPPPEYNVCLGDLFSVAWLEDSDVQNSWYETLNQQYHHVDKRISHASHATQYGNLKLGEEGLFVYMGSNPANDNYTSLDGNALTPSSIVVNQRDAD-


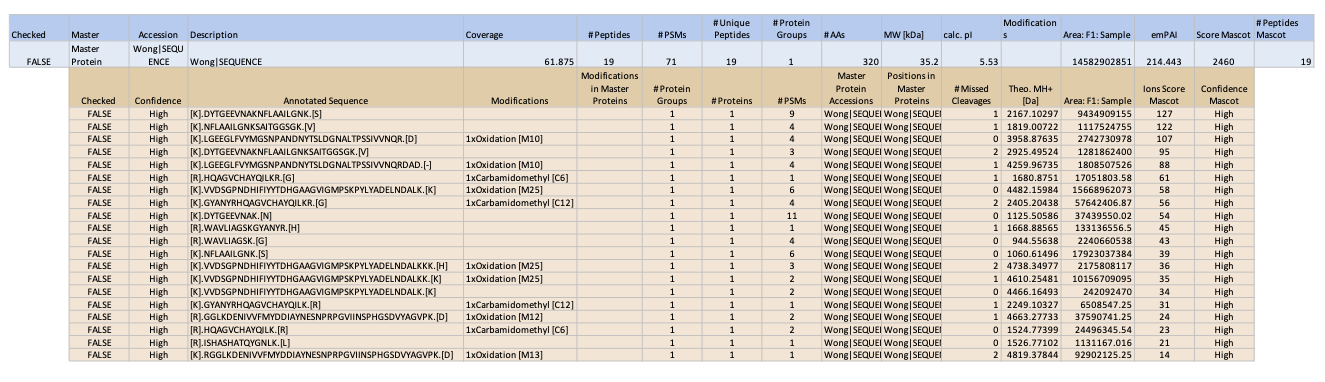


**Table S1: MASCOT sequence query results of purified *Oa*AEP1b-C247A Δ351**. Purified protein was trypsin digested and each digested fragments were analyzed via mass spectrometry. The mass of each fragment was then blasted against the known sequences of *Oa*AEP1b-C247A using MASCOT program.
